# Functional delineation of rice *MADS29* reveals its role in embryo and endosperm development by affecting hormone homeostasis

**DOI:** 10.1093/jxb/ert231

**Published:** 2013-08-08

**Authors:** Saraswati Nayar, Rita Sharma, Akhilesh Kumar Tyagi, Sanjay Kapoor

**Affiliations:** ^1^Interdisciplinary Centre for Plant Genomics and Department of Plant Molecular Biology, University of Delhi South Campus, Benito Juarez Road, New Delhi 110021, India; ^2^National Institute for Plant Genome Research, Aruna Asaf Ali Marg, New Delhi 110067, India; *Present address: Department of Plant Pathology, University of California, Davis, CA 95616, USA

**Keywords:** Cytokinin, embryo, endosperm, MADS box, *Oryza sativa*, starch, seed development.

## Abstract

Rice MADS29 has recently been reported to cause programmed cell death of maternal tissues, the nucellus, and the nucellar projection during early stages of seed development. However, analyses involving OsMADS29 protein expression domains and characterization of OsMADS29 gain-of-function and knockdown phenotypes revealed novel aspects of its function in maintaining hormone homeostasis, which may have a role in the development of embryo and plastid differentiation and starch filling in endosperm cells. The *MADS29* transcripts accumulated to high levels soon after fertilization; however, protein accumulation was found to be delayed by at least 4 days. Immunolocalization studies revealed that the protein accumulated initially in the dorsal-vascular trace and the outer layers of endosperm, and subsequently in the embryo and aleurone and subaleurone layers of the endosperm. Ectopic expression of MADS29 resulted in a severely dwarfed phenotype, exhibiting elevated levels of cytokinin, thereby suggesting that cytokinin biosynthesis pathway could be one of the major targets of OsMADS29. Overexpression of *OsMADS29* in heterologous BY2 cells was found to mimic the effects of exogenous application of cytokinins that causes differentiation of proplastids to starch-containing amyloplasts and activation of genes involved in the starch biosynthesis pathway. Suppression of *MADS29* expression by RNAi severely affected seed set. The surviving seeds were smaller in size, with developmental abnormalities in the embryo and reduced size of endosperm cells, which also contained loosely packed starch granules. Microarray analysis of overexpression and knockdown lines exhibited altered expression of genes involved in plastid biogenesis, starch biosynthesis, cytokinin signalling and biosynthesis.

## Introduction

Hormones play a crucial role in regulating development of seeds, starting from early embryo and endosperm patterning till its maturation (Sun *et al*., 2010). In cereals, cytokinins are required during early endosperm development (5–10 days after pollination (DAP) in maize, 6–15 DAP in rice) where their presence coincides with active cell division, which also corresponds to the grain filling stage (Lur and Setter, 1993; Yang *et al*., 2000, 2002). Auxin accumulation, however, peaks at a later stage (first peak at 9–11 DAP, second peak at 20 DAP), when cells in the endosperm and the embryo enter the phase of differentiation (Lur and Setter, 1993). At later stages of seed development, the levels of auxin and cytokinin decline, leading the way for the maturation- and dormancy-related hormones, abscisic acid, and gibberellic acid (White *et al*., 2000).

During grain filling, sugars are translocated from vegetative tissues to the developing endosperm, converted to amylose and amylopectin, and stored in the form of starch granules in modified plastids called amyloplasts (Thomson and Whatley, 1980; Sonnewald and Kossmann, 2013). The molecular aspects of differentiation of starch-storing amyloplasts from the undifferentiated proplastids are largely unknown, although cytokinins have been shown to be involved in the process of grain filling in cereals (Yang *et al*., 2000). Major work on proplastid differentiation and the role of hormone homeostasis therein has been carried out in the tobacco BY-2 cell line, which is maintained in a medium containing auxin (2-4D). Upon addition of cytokinin (6-benzylaminopurine) to the medium, proplastids in BY-2 cells differentiate into amyloplasts and develop starch granules (Miyazawa *et al*., 2002). The process of plastid differentiation is accompanied by induction of several nucleus-encoded plastid genes related to starch biosynthesis, such as *ADP-glucose pyrophosphorylase* (*AgpS)* and *GRANULE BOUND STARCH SYNTHASE* (*GBSS*) (Miyazawa *et al*., 1999). Cytokinins have also been shown to regulate plastid biogenesis and function in *Lupinus luteus*, maize, and tobacco (Kusnetsov *et al*., 1998; Polanska *et al*., 2007; Brovko *et al*., 2010).

MADS-box transcription factors are homeotic genes that have primarily been associated with flower development (Kater *et al*., 2006). However, some MADS-box genes were also predicted to play roles in regulation of seed development in rice (Arora *et al*., 2007). In *Arabidopsis*, two MIKC^C^ B-sister MADS-box genes, *ABS/TRANSPARENT TESTA16* (*TT16*) and *GORDITA* (*AGL63*), have been shown to play a role in seed and fruit development. *ABS/TT16* knockout results in the absence of the endothelial layer, which results in seeds lacking proanthocynadin accumulation, thereby affecting seed coat colour without any effect on seed set (Nesi *et al*., 2002). *GORDITA* is another B-sister gene in *Arabidopsis* and has a role in maintaining cell size of the outer integument in the seed and additional role in repressing fruit growth (Prasad *et al*., 2010). *FBP24* closely resembles the *Arabidopsis ABS/TT16*, and its knockout in petunia results in complete ablation of the endothelial layer leading to a lower seed set (de Folter *et al*., 2006). Furthermore, a MIKC^C^ B-sister gene (*Wheat B-sister*, *WB*_*SIS*_), along with a D-class gene (*SEEDSTICK*, *STK*), has been shown to be associated with ectopic ovule formation in the pistil-like stamens of alloplasmic wheat CMS line, which exhibits pistillody (development of incomplete ectopic ovules from the pistil-like stamens) (Yamada *et al*., 2009). Taken together, these reports suggest the involvement of the B-sister class of MADS in ovule and seed/fruit development in both dicots and monocots.

*OsMADS29* is a MIKC^C^ class type II MADS-box gene that, with two other MADS-box genes, *OsMADS30* and *OsMADS31*, belongs to the B-sister subclade in rice (Arora *et al*., 2007). This gene is expressed in seeds (Arora *et al*., 2007) and has been implicated in endosperm development by means of programmed cell death-driven degeneration of maternal as well as filial tissues (Yang *et al*., 2012; Yin and Xue, 2012).

In the present study, a combination of microarray, qPCR, Western, and immunolocalization analyses were carried out to determine the precise spatiotemporal domains of *OsMADS29* expression. The analysis of the *MADS29* gain-of-function phenotype highlighted its important role in regulating hormone homeostasis. The ability of *OsMADS29* to mimic the effects of exogenously added cytokinins in the heterologous tobacco BY-2 cells indicated that the *OsMADS29* targets, which influence cytokinin biosynthesis, exist in both dicots and monocots. The RNAi-based knockdown phenotype highlighted its role in endosperm and embryo development. Taken together, the results suggest that *MADS29* plays a role in multiple aspects of seed development, including plastid biogenesis, starch biosynthesis, cell division, and differentiation in both embryo and endosperm by affecting cytokinin and auxin homeostasis in target cell types. A comparison of the transcript and protein accumulation patterns show that the expression of *OsMADS29* is tightly regulated at the transcriptional as well as the post-transcriptional level.

## Materials and methods

### Plant material and growth conditions

Wild-type (WT) and transgenic (*Oryza sativa* subsp. indica var. PB1) rice plants were grown in soil/vermi-compost/organic compost mix (3:1:2) supplemented with NPK in the growth chamber with a 14/10 light/dark cycle at 30/28 °C until tillering, and then, to facilitate flowering, with a 12/12 light/dark cycle at 28/26 °C. Leaves and seeds were collected, frozen in liquid nitrogen, and stored at –70 °C until further use. For immunolocalization, protein isolation, and RNA isolation, rice plants (*O. sativa* subsp. indica var. IR64) were grown at experimental fields of IARI (Pusa, New Delhi) in kharif season (from mid-June to September; T_max_ 35–40 °C; T_min_ 25–29 °C).

### Preparation of constructs and plant transformation

Rice transgenics for *MADS29* knockdown and overexpression lines and BY2 transgenics overexpressing *MADS29* were generated. A detailed description of constructs and plant transformation are described in Supplementary Methods S1 (available at *JXB* online).

### Western blot analysis and immunolocalization of MADS29

Protein was extracted from S1–S5 seed stages, P1–P6 panicle stages, mature leaf, mature root, and germinating seeds (0–120h after imbibition). For detailed description of protein extraction, Western blot analysis, and immunolocalization, refer to Supplementary Methods S1.

### Morphological and anatomical characterization of rice transgenics

*MADS29*-overexpression (MADS29^OX^) plants were photographed using a digital camera (EOS 1000, Canon, Japan). Florets of MADS29^OX^ and seeds of RNAi-knockdown (MADS29^KD^) were dissected and photographed using a stereozoom microscope (MZ12.5, Leica, Wetzlar, Germany). Lamina joints of WT and MADS29^OX^ and the mature embryo regions of MADS29^KD^ and WT seeds were fixed in formaldehyde/acetic acid/ethanol (3.7:5:50) overnight, dehydrated in a graded ethanol series (30–100%) followed by a graded tertiary-butanol series (25–100%), and embedded in Paraplast Plus (Sigma Aldrich). Sections (4–6 µm thick) were cut using a Leica RM2245 rotary microtome and were stained with 1% toluidine blue, mounted in distyrene plasticizer xylene, and observed under a compound microscope (DM 5000B, Leica). Seed morphology and endosperm characteristics were observed under a scanning electron microscope (EvoLS25, Zeiss, Germany) as described previously (She *et al*., 2010).

### Visualization of starch granules in BY2 transgenic cell lines

To study the morphological characters of the transgenic and WT BY2 cells, a drop of the unstained or stained (Lugol’s solution; 5%, w/v, I_2_ in 10% KI) 3-day-old cell culture (grown in Linsmaier Skoog medium containing 0.2mg/l 2,4-D) was spotted on a slide and observed using DIC optics (DM 5000B, Leica). BY2 cells expressing *MADS29* were subjected to different concentrations of 2,4-D (0.4 and 0.6mg/l) for 3 days and observed under the microscope (DM 5000B, Leica) and photographs were taken using the digital camera attached to it.

### RNA isolation

For details regarding RNA isolation, refer to Supplementary Methods S1.

### Transcriptome analysis of MADS29^OX^ and MADS29^KD^ plants

Microarray of *MADS29* knockdown lines and overexpression lines was performed according to the Affymetrix protocol. For further details of the microarray experiment and analysis, refer to Supplementary Methods S1. The microarray data have been deposited into the Gene Expression Omnibus database (GSE42029 and GSE42028; http://www.ncbi.nlm.nih.gov/geo).

### Quantitative PCR analysis

Quantitative PCR (qPCR) reactions and conditions were carried out as described by Arora *et al*. (2007). For further details, refer to Supplementary Methods S1 and Supplementary Table S4.

### Phylogenetic analysis

Phylogenetic analysis for MADS29 and its related members was carried as described in Supplementary Methods S1.

### Hormone extraction and ultra-high-performance chromatography

For details regarding hormone extraction and ultra-performance liquid chromatography (UPLC), refer to Supplementary Methods S1.

## Results

### *OsMADS29* phylogenetic and expression analyses

**Fig. 1.**
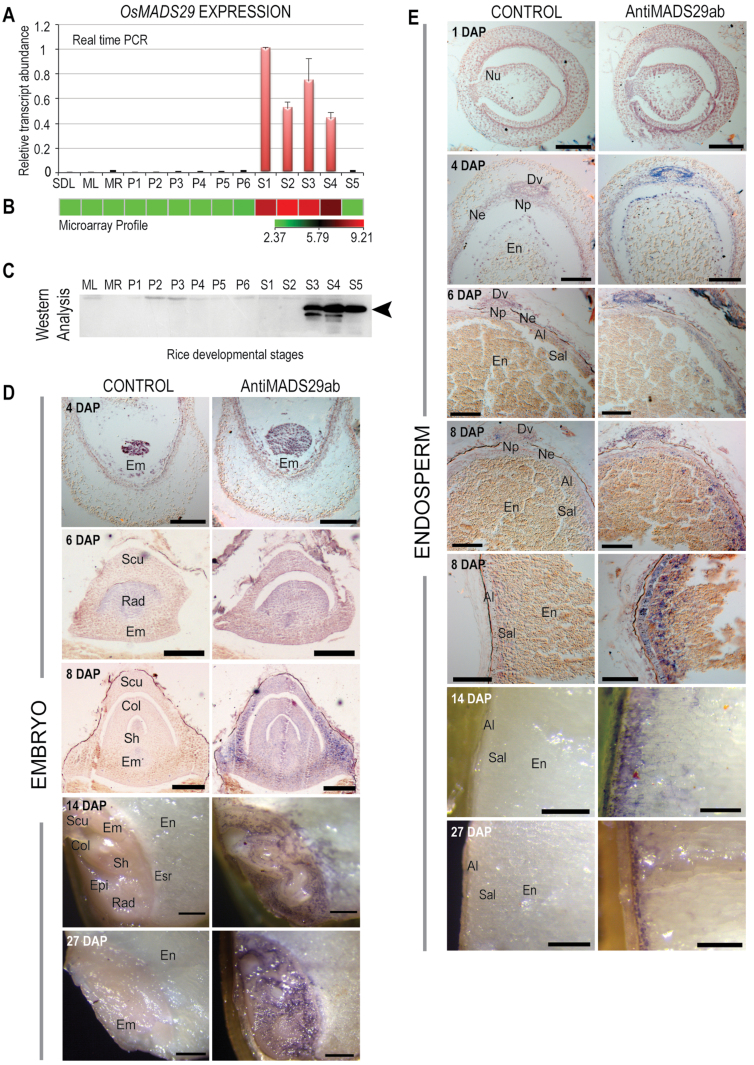
Temporal and spatial expression of MADS29 across developmental stages of rice. (A) Relative transcript levels of *MADS29* using real-time PCR; bars indicate standard error (*n* = 3). (B) Microarray-based expression profile of MADS29; bar indicates log_2_ expression values. (C) Western blot analysis of MADS29 using MADS29 antibody across rice developmental stages. ML, mature leaf; MR, mature root; P1–P6, panicle stages; S1–S5, seed stages; SDL, seedling. (D) Immunolocalization of MADS29 expression in the developing embryo at 4, 6, 8, 14, and 27 days after pollination (DAP); bars: 100 µm (4, 6, and 8 DAP), 400 µm (14 and 27 DAP). (E) Immunolocalization of MADS29 expression in the developing endosperm at 1, 4, 6, 8, 14, and 27 DAP. The nucellar region and the aleurone region at 8 DAP are shown separately: bars: 100 µm (4, 6, and 8 DAP), 500 µm (14 and 27 DAP). Al, aleurone; Col, coleoptile; Dv, dorsal vascular bundle; Em, embryo; En, endosperm; Epi, epiblast; Esr, embryo-surrounding region; Ne, nucellar epidermis; Np, nucellar projection; Nu, nucellus; Rad, radicle; Sal, subaleurone; Scu, scutellum; Sh, shoot.

*OsMADS29* (LOC_Os02g07430) codes for a putative B-sister-type MIKC^C^ transcription factor, which, along with its orthologues in *Zea mays* (ZMM17; Becker *et al*., 2002), *Sorghum bicolor* (SbB-SISTER, NCBI accession ref|XP_002453370.1|), *Triticum aestivum* (TaAGL35; Yamada *et al*., 2009), and *Hordeum vulgare* (HvB-SISTER, NCBI accession dbj|BAK06913.1|), forms a distinct monocot-specific subgroup (Supplementary Fig. S1). *OsMADS29* transcripts have been shown to accumulate specifically in seeds from the day of anthesis to up to 20 DAP, corresponding to S1–S4 stages of seed developmental (Lee *et al*., 2003; Arora *et al*., 2007; Yin and Xue, 2012). The seed development stages correspond to 0–2 (S1), 3–4 (S2), 5–10 (S3), 11–20 (S4), and 21–29 DAP (S5), respectively (Sharma *et al*., 2012). The expression meta-analysis, however, revealed *OsMADS29* expression also in the gynoecium in pre-anthesis spikelets (ROAD; http://www.ricearray.org/expression/meta_analysis.shtml). The *OsMADS29* expression was validated using qPCR, where the results were found to be in complete agreement with this group’s previously described microarray results (Lee *et al*., 2003; Arora *et al*., 2007; Yin and Xue, 2012), where high levels of *OsMADS29* transcripts were found to accumulate during S1–S4 stages of seed development, with almost undetectable expression in vegetative and panicle tissues (Fig. 1A, B). The absence of *MADS29* transcripts from the pre-anthesis-stage gynoecium might be either due to restricted expression in specific cell types of the gynoecium or varietal differences, or both.

*OsMADS29* is predicted to code for a 260-amino-acid polypeptide with a calculated molecular weight of 28.99kDa (Arora *et al*., 2007). Anti-MADS29 antibodies raised against an 11-residue MADS29-specific synthetic peptide were used to profile the OsMADS29 protein levels across vegetative (seedling, mature leaf, mature root), panicle (P1–P6), and seed (S1–S5) developmental stages corresponding to those used for transcript analysis. Surpringly, OsMADS29 accumulated at very low (almost undetectable) levels in stages S1 and S2 (0–4 DAP) of seed development, which showed highest accumulation of the *OsMADS29* transcripts. However, significantly high levels of the OsMADS29 polypeptide became detectable from the S3 stage (5–10 DAP) onwards. The protein was observed close to the 28-kDa marker band, thus confirming its predicted molecular weight and its specificity to seed developmental stages (Fig. 1C.)

With *in situ* hybridization, the *MADS29* transcripts have earlier been shown to accumulate in the nucellus and the nucellus projection in early stages of seed development and in the developing as well as the mature embryo (Yin and Xue, 2012). To gain insight into the spatiotemporal accumulation pattern of the MADS29 protein, transverse sections of developing seed from representative days after pollination (corresponding to S1–S5 stages) were subjected to immunolocalization analysis using anti-MADS29 antibody. At 1 DAP (S1) the MADS29 protein was not detectable, but at 4 DAP (S2) a faint signal was detected around the dorsal vascular trace, the nucellar epidermis region, and the outer cell layer of the developing endosperm (Fig. 1E). Although Yin and Xue (2012) reported maximum accumulation of *MADS29* transcripts in the nucellus at 0 and 1 DAP, this study did not find any protein expression matching the transcript level at these stages using both Western blot and immunolocalization analyses. Moreover, the protein was conspicuously missing from the nucellar projection region (Supplementary Fig. S2). During developmental progression from S2 to S3, the MADS29 protein content further declined in the nucellar region, while a concomitant and gradual increase in its accumulation was observed in the embryo, aleurone, and subaleurone layers (Fig. 1D, E 14 and 27 DAP). At the S5 stage, the level of protein decreased in the subaleurone layer and expression was confined to the aleurone in the endosperm (Fig. 1E 27 DAP).

### Ectopic expression of *OsMADS29* shifts hormonal balance in favour of cytokinins

**Fig. 2.**
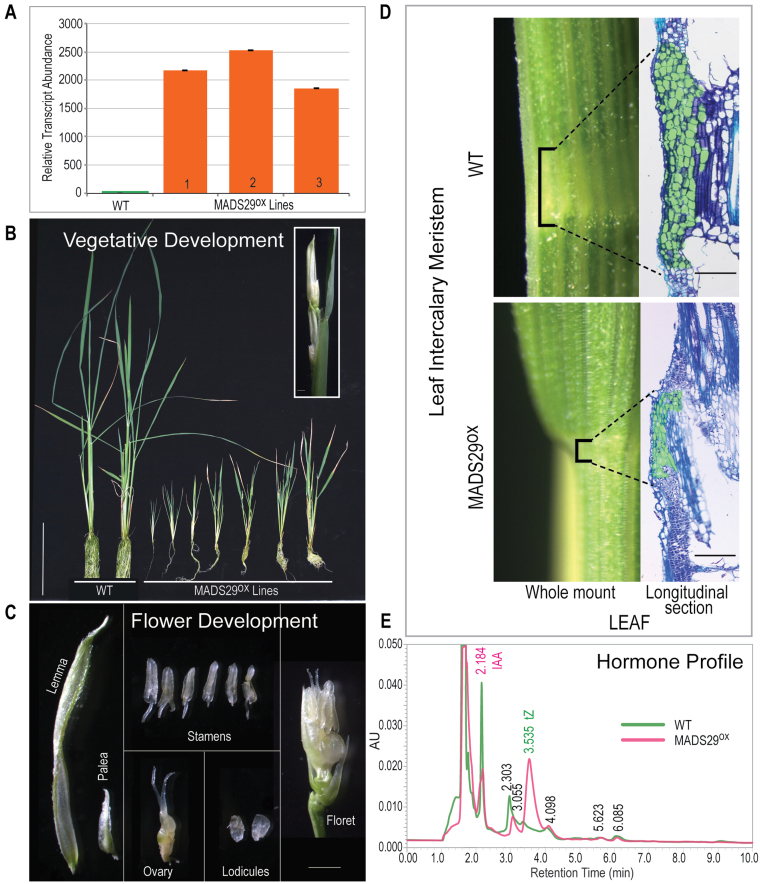
Characterization of the *MADS29* overexpression (MADS29^OX^) phenotype in transgenic rice. (A) Relative transcript abundance of MADS29 in MADS29^OX^ lines; bars indicate standard error (*n* = 3). (B) Morphology of MADS29^OX^ lines; inset shows an emerging panicle; bar: 5cm. (C) Floral organs of a single MADS29^OX^ floret; bar: 1mm. (D) Comparison of intercalary meristematic zone in wild-type and MADS29^OX^ leaf; bar: 100 µm. (E) Hormone (auxin/cytokinin) profile in leaves of wild-type and MADS29^OX^ plants; tZ, *trans-*zeatin; IAA, indole acetic acid.

Rice var. PB1 transformation with pUBI:OsMADS29cDNA: NosT yielded very few transgenic plants because of low regeneration frequency. The upregulation of *MADS29* in the leaf samples of hygromycin-resistant PCR-positive transformant lines was confirmed by qPCR, which was found to be at least 1800-fold higher than in the WT (Fig. 2A). MADS29^OX^ plants were severely stunted and failed to produce any seeds. Also, the leaves in MADS29^OX^ plants were much thinner and smaller than the WT and root development was significantly affected; however, leaf number in these plants was not affected because the plants developed 9–10 miniaturized leaves (Fig. 2B). Flowering in these plants was early (~45 days) and the panicle in each plant consisted of three or four florets (Fig. 2B). As for flower organs, the size of the palea was significantly reduced, the pistil was deformed (appeared slightly swollen and the stigma was glabrous due to loss of its feathery appearance), and the anthers were not fertile because of the absence of pollen grain; however, the lodicules appeared normal (Fig. 2C). Hence, the florets were sterile and failed to produce any seeds. As all the floral whorls in the MADS29^OX^ floret were intact, it suggested that overexpression of *MADS29* does not majorly affect floral organ identity; instead, it caused aberration in the overall morphology and fertility of the flower (Fig. 2C).

There is a growing body of evidence which suggests that the antagonistic interaction of auxin and cytokinin in the meristematic regions directly affects plant growth and development by regulating number of undifferentiated cells in the root and shoot apical meristems (Su *et al*., 2011). In the root meristem, auxin induces cell division, whereas cytokinin promotes differentiation (Dello Ioio *et al*., 2008; Moubayidin *et al*., 2009). In contrast, in the shoot meristem, cytokinin promotes proliferation of undifferentiated stem cells, while auxin triggers differentiation of organ primordia (Su *et al*., 2011). The ratio of cytokinin and auxin, therefore, is crucial in maintaining the balance between cell division and rate of differentiation to sustain development.

To determine if the reduction in the size of vegetative organs in MADS29^OX^ plants was associated with meristem size, the leaf intercalary meristem regions in WT and MADS29^OX^ plants were compared. As shown in Fig. 2D, the number of undifferentiated stem cells in the intercalary meristem in MADS29^OX^ plants was significantly reduced.

To determine, if the stunted phenotype and reduced size of intercalary meristem in the MADS29^OX^ lines were associated with an imbalance in the auxin/cytokinin ratio, the levels of *trans*-zeatin riboside, *trans*-zeatin (tZ), and indole-3-acetic acid (IAA) were measured using UPLC from 50mg leaf tissue of WT and MADS29^OX^ plants. The retention times of the corresponding standards (tZ, *trans*-zeatin ribosides, and IAA) were used as reference. The results, as shown in Fig. 2E, confirmed the reduction in IAA levels by more than 2-fold, and a significant increase (>5-fold) in the levels of tZ levels in MADS29^OX^ leaves, thereby shifting the hormonal balance in favour of cytokinins. To check if exogenous application of auxin could restore the MADS29^OX^ phenotype, IAA (0.2–0.8mg/l) was exogenously supplied. MADS29^OX^ plants responded to the auxin application, especially at 0.4mg/l, by initiation of numerous root hairs and inhibition of lateral root formation; however, it did not rescue the phenotype. The result, however, indicated that auxin perception and its signalling was not affected as a result of *MADS29* overexpression (Supplementary Fig. S3).

### *OsMADS29* overexpression mimics the cytokinin over-dosage response in tobacco BY2 cells

**Fig. 3.**
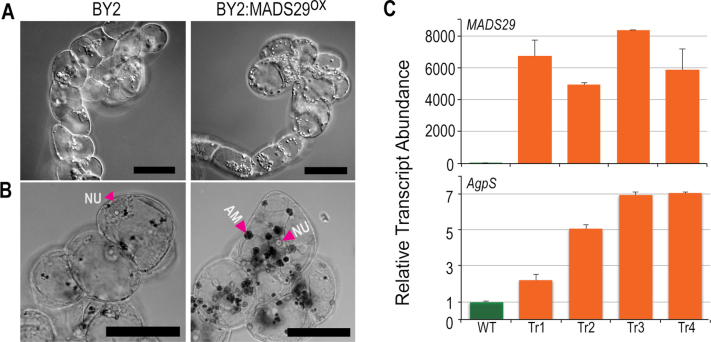
*OsMADS29* expression in BY2 cells. (A) BY2:MADS29^OX^ stable transgenic cells showing development of multiple organelles under DIC mode; bar: 50 µm. (B) I_2_KI staining of BY2 cells to detect amyloplasts: AM, amyloplast; NU, nucleus; bars, 50 µm. (C) qPCR analysis to determine *MADS29* and *AgpS* transcript levels in wild-type BY2 and transgenic BY2:MADS29^OX^ lines; bars indicate standard error (*n* = 3) (this figure is available in colour at *JXB* online).

Since a high cytokinin-led reduction in intercalary meristem and overall reduction in growth in MADS29^OX^ rice transgenic lines was observed, cytokinin metabolism was thought to be one of the main targets of MADS29. In previous studies, exogenous application of high cytokinin concentrations was shown to cause differentiation of undifferentiated proplastids to amyloplasts in the tobacco BY2 cell line (Sakai *et al*., 1992; Miyazawa *et al*., 1999, 2002). To test if the gain-of-function MADS29^OX^ phenotype manifested because MADS29 could influence the universally existing cytokinin metabolism pathway outside its expression domain (which, in the case of rice, is seed development), tobacco BY2 cells were stably transformed with a *MADS29*-overexpression construct. Mimicking the effect of exogenously added cytokinin, BY2:MADS29^OX^ cells exhibited an uncharacteristically high number of enlarged amyloplasts in comparison to untransformed BY2 cells (Fig. 3A). To confirm if the observed organelles were indeed amyloplasts, cells were stained with Lugol’s solution (Miyazawa *et al*., 1999). Blue-black staining of starch grains in these organelles established that these were indeed amyloplasts (Fig. 3B). The expression of *OsMADS29* in four BY2 transgenic calli was confirmed by qPCR (Fig. 3C upper panel). Furthermore, as excess of auxin was shown to reverse the effects of excess cytokinins (Miyazawa *et al*., 1999, 2002), exogenous addition of increasing concentrations of auxin (0.4 and 0.6mg/l) led to the inhibition of amyloplast development (Supplementary Fig. S4). As previously evidenced, high levels of cytokinin triggered accumulation of transcripts of key starch metabolism enzymes such GBSS, AgpS, and starch branching enzyme in BY2 cells (Miyazawa *et al*., 1999, 2002). The qPCR analysis confirmed a 2–7-fold increase in *AgpS* transcript levels in the BY2:MADS29^OX^ lines in comparison to untransformed BY-2 cells (Fig. 3C lower panel).

### MADS29 has a vital role in endosperm and embryo development: analysis of the knockdown phenotype

**Fig. 4.**
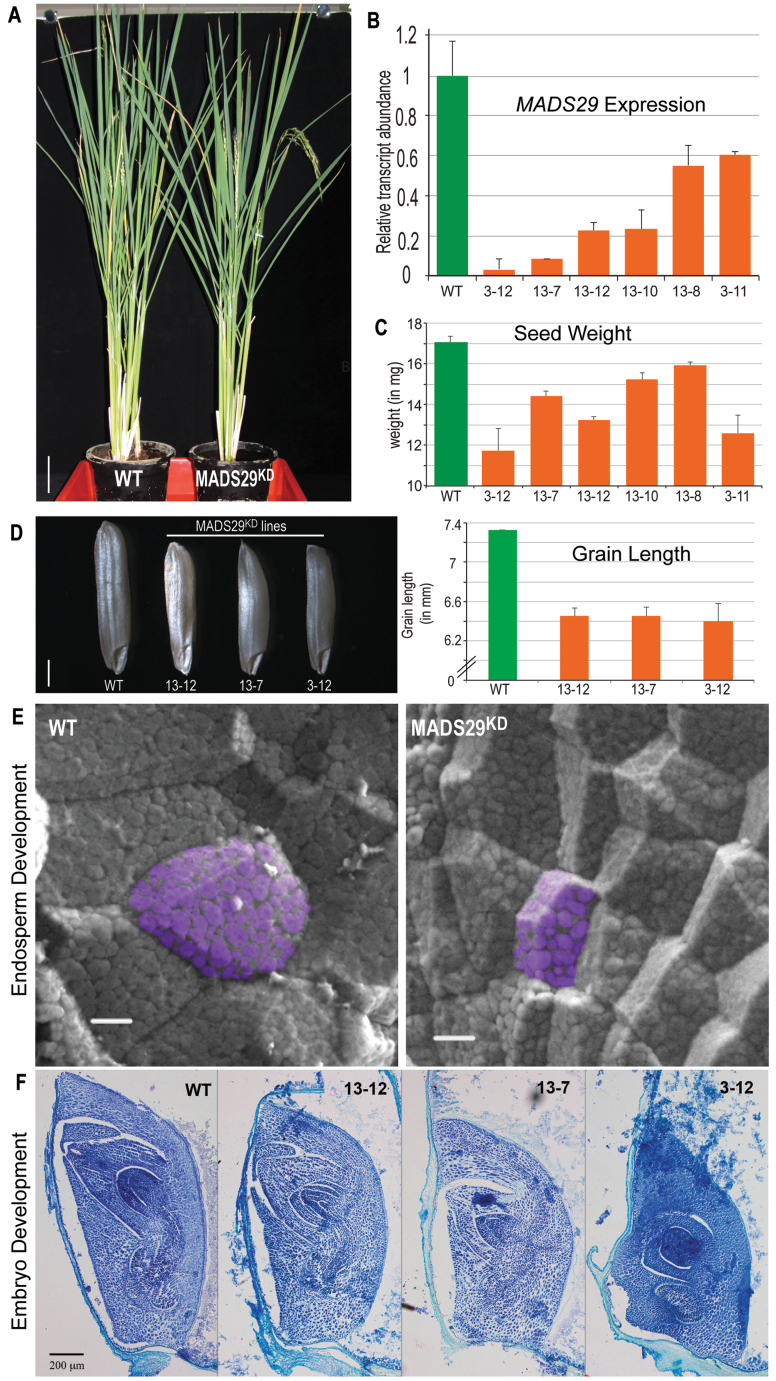
RNAi-mediated *OsMADS29* knockdown phenotype. (A) Comparison of wild-type and MADS29^KD^ vegetative development; bar: 7cm. (B) *OsMADS29* transcript accumulation in wild-type and MADS29^KD^ lines as determined by qPCR; bars indicate standard error (*n* = 3). (C) Comparison of dry seed weight between wild-type and MADS29^KD^ lines; bars indicate standard error (*n* = 45). (D) Left panel: representative mature seeds from wild-type and three MADS29^KD^ lines, bar: 1mm; right panel: comparison of grain length between wild-type and MADS29^KD^ lines where *MADS29* silencing results in decreased length of mature grain; bars indicate standard error (*n* = 20). (E) Scanning electron micrographs of half-split endosperms from wild-type and MADS29^KD^ plants showing organization of cells and sizes and structures of starch granules. Starch granules in representative cells have been pseudocoloured purple using Adobe Photoshop; bar: 10 µm. (F) Toluidine-blue-stained longitudinal sections of embryos from wild-type and MADS29^KD^ lines showing reduction in size and developmental abnormalities in the MADS29^KD^ lines; bar, 200 µm.

Previous studies by Yin and Xue (2012) and Yang *et al*. (2012) have shown that MADS29 has an active role during early seed development by regulating programmed cell death of maternal as well as filial tissues. Investigation of the MADS29^KD^ rice transgenic lines revealed that there was no apparent morphological difference between MADS29^KD^ and the WT during vegetative development and flowering (Fig. 4A); however, in mature panicles, the percentage of filled grains were significantly reduced in the MADS29^KD^ lines. Real-time qPCR confirmed a significant reduction (40–90%) in *MADS29* endogenous transcript levels (Fig. 4B). Lines 3–12, 13–7, and 13-12 were chosen for further characterization as they exhibited the highest degree of *MADS29* silencing. This study observed a reduction (approximately 7–30%) in the dryweight of mature seeds, which correlated with reduction in grain size (Fig. 4C). In these lines, mature seeds appeared shrunken, indicating decreased grain filling, and also the seed length was reduced by approximately 13% (Fig 4D). The reduction in the key grain characteristics such as grain weight and grain length associated with a decrease in grain size, as observed in the *flo2* mutant grains (She *et al*., 2010). Scanning electron microscopy of the half-cut seeds indicated that in the MADS29^KD^ lines the endosperm was filled with loosely packed, small, and spherical starch granules with large spaces in between, whereas the WT endosperm consisted of densely packed, large, and polyhedral starch granules (Fig. 4E). The endosperm cells were also smaller in the MADS29^KD^ lines in comparison to the WT. Longitudinal embryo sections were analysed to observe any defects in embryo development. Embryo size was reduced in the knockdown lines, and line 3–12 showed the most severe phenotype, correlating with a maximum reduction in *MADS29* transcript, followed by lines 13–7 and 13-12 (Fig. 4F), which supports the result obtained by Yang *et al*. (2012). These results point to *MADS29*’s plausible role in amyloplast development, grain filling, and cell division in the endosperm and development of embryo.

### Transcriptome analysis of *OsMADS29* overexpression and knockdown plants to identify downstream pathways

**Fig. 5.**
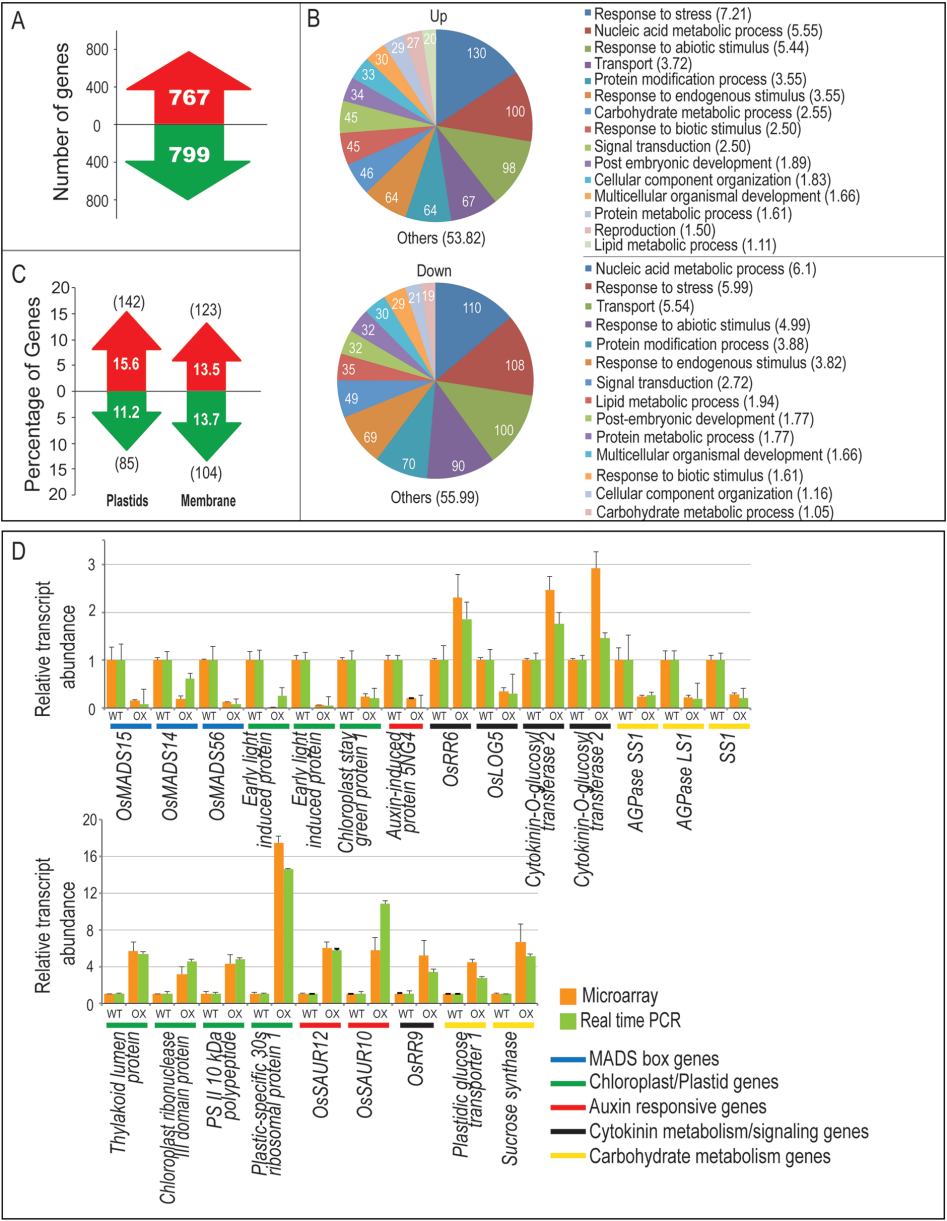
Microarray-based comparison of MADS29^OX^ and WT leaf transcriptomes. (A) Total number of 2-fold upregulated genes (red) and downregulated genes (green) in MADS29^OX^ leaf samples in comparison to the WT (*P* ≤ 0.05, *n* = 3). (B) Functional categorization of up- and downregulated genes into biological process GO classes in MADS29^OX^ leaves; values on the pie chart and in parentheses are percentage and number of genes represented in each category; categories with <1% share of the differentially expressed genes are cumulatively represented in the ‘Others’ category. (C) Proportion of differentially expressed genes included in two major ‘cellular component’ GO categories, ‘membrane’ and ‘plastids’; values in parentheses show the number of genes; red and green arrows depict up and downregulation, respectively. (D) Real-time quantitative PCR validation of selected genes from the highlighted pathways; transcript profiles of low- and high-abundance transcripts are shown in upper and lower panels, respectively; bars indicate standard error (*n* = 3) (this figure is available in colour at *JXB* online).

The 3rd and 4th leaves from the flag leaf in MADS29^OX^ and WT rice var. PB1 plants were subjected to microarray-based transcriptome analysis to identify the downstream genes and pathways affected because of the ectopic expression of *MADS29*. A total of 1566 genes were differentially expressed (≥2-fold change at *P* ≤ 0.05: 767 genes were upregulated and 799 were downregulated (Fig. 5A). The major biological process GO categories highlighted in the MADS29^OX^ differentially expressed genes included stress, nucleic acid metabolism, transport, protein modification process, and response to endogenous stimulus (Fig. 5B). Another interesting observation was that, under the cellular component GO classification, plastid genes (15.6%) and membrane genes (13.74%), and associated genes, constituted the major up- and downregulated categories, respectively (Fig. 5C).

As there seemed to be a cytokinin/auxin imbalance in the MADS29^OX^ plants, the MADS29^OX^ leaf microarray data were compared to an earlier reported microarray dataset of tZ-treated rice leaf samples (GSE6737; Hirose *et al*., 2007). In tZ-treated leaves, a total of 4179 genes were differentially expressed by ≥2-fold (*P* ≤ 0.05). Interestingly, 506 genes were common between MADS29^OX^ and tZ-treated rice plants, of which 211 genes were commonly downregulated, 137 genes were commonly upregulated, and 158 genes showed reciprocal expression patterns. This implied that almost 32% of the differentially regulated genes in MADS29^OX^ lines were cytokinin responsive (Supplementary Fig. S5).

**Fig. 6.**
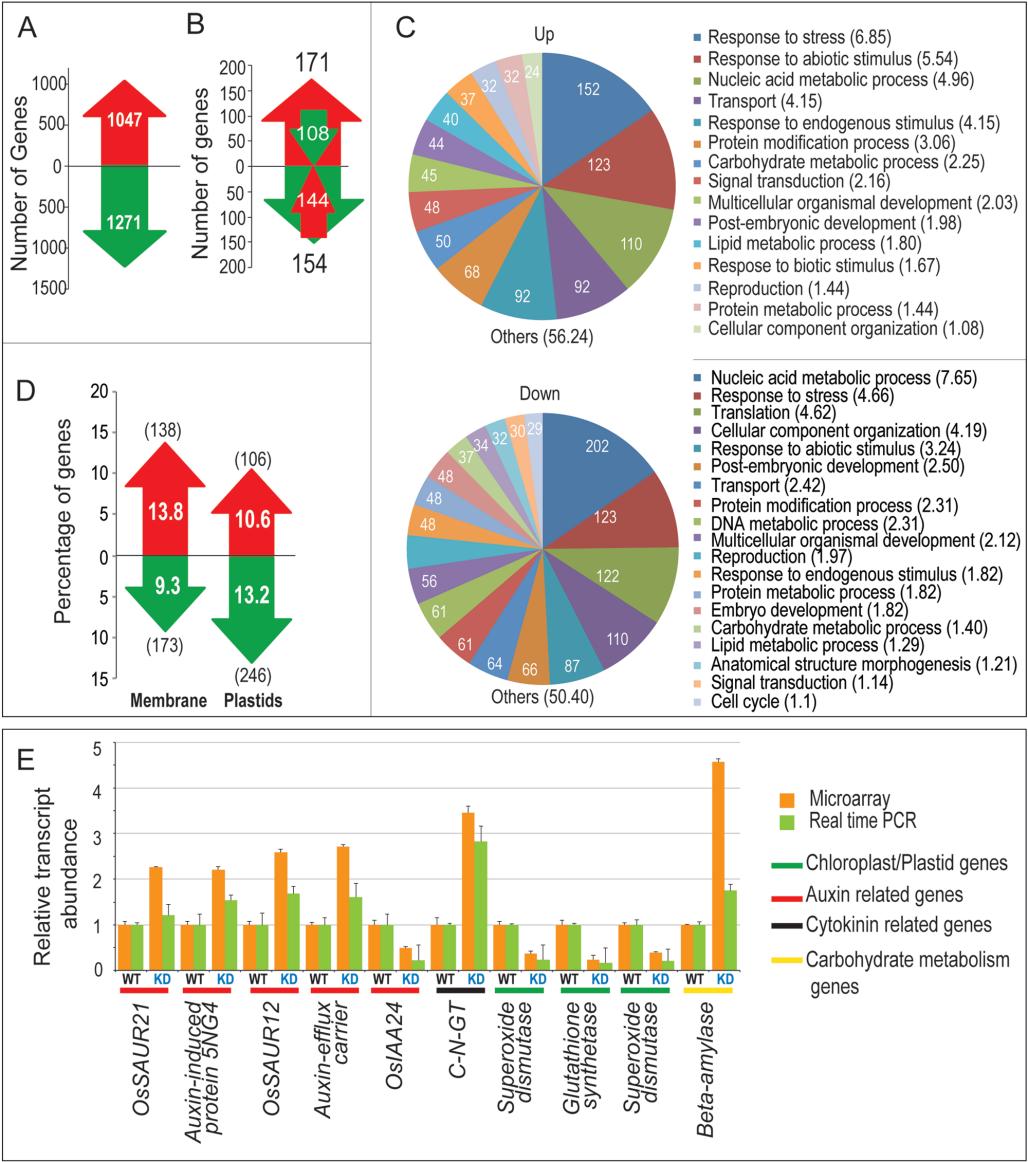
Comparison of MADS29^KD^ and wild-type S3-stage seed transcriptomes. (A) Total number of 2-fold upregulated genes (red) and downregulated genes (green) in MADS29^KD^ seed samples in comparison to the WT (*P* ≤ 0.05, *n* = 3). (B) Differentially expressed genes showing inverse correlation between the MADS29^OX^ lines (large arrows) and the MADS29^KD^ lines (inset arrows). (C) Functional categorization of up- and downregulated genes into biological process GO classes in MADS29^KD^ seeds; values on the pie chart and in parentheses are percentage and number of genes represented in each category; categories with <1% share of the differentially expressed genes are not shown individually but are included in the ‘Others’ category. (D) Proportion of differentially expressed genes included in two major ‘cellular component’ GO categories, ‘membrane’ and ‘plastids’; values in parentheses show the number of genes; red and green arrows depict up- and downregulation, respectively. (E) Real-time quantitative PCR validation of microarray data for selected genes from the highlighted pathways; bars indicate standard error (*n* = 3) (this figure is available in colour at *JXB* online).

To identify downstream genes affected by *MADS29* silencing, a genome-wide transcriptome analysis was undertaken for S3-stage seeds (the first stage to show significant MADS29 protein accumulation) in MADS29^KD^ and WT plants. Both microarray and qPCR analyses confirmed a 4-fold downregulation of *MADS29* expression in MADS29^KD^ developing seeds. A total of 2318 genes were found to be differentially expressed by >2-fold between MADS29^KD^ and WT (*P* < 0.05). Of these, 1271 genes were downregulated and 1047 genes were upregulated (Fig. 6A). Of these, 325 genes were common to the lists of differentially expressed genes in the MADS29^OX^ lines. A large proportion of these common differentially expressed genes also exhibited reciprocal expression patterns in the MADS29^KD^ and MADS29^OX^ lines. Of the 171 genes upregulated in MADS29^OX^, 108 were correspondingly downregulated in MADS29^KD^, whereas out of 154 that were downregulated in MADS29^OX^, 144 were upregulated in MADS29^KD^ plants, indicating mutual reciprocity of gene expression patterns in overexpression and knockdown plants (Fig. 6B). GO analysis based on biological processes revealed over-representation of genes involved in stress and nucleic acid metabolism in both the up- and downregulated categories (Fig. 6C). Genes involved in transport and response to endogenous stimuli were among the major categories of upregulated genes, whereas genes related to translation and cellular component organization comprised the major downregulated categories in MADS29^KD^ lines. Furthermore, similar to that in MADS29^OX^ plants, a significant percentage of differentially expressed genes were associated with plastid and membrane compartments. Interestingly, for the upregulated genes in MADS29^KD^ lines, cellular component GO terms associated with membrane were maximally represented (13.83%), and for downregulated genes, plastid-related GO terms formed the top category (13.16%) (Fig. 6D). The opposite of this was true in MADS29^OX^ lines, thus indicating a role for MADS29 in plastid differentiation.

This study selected 23 differentially expressed genes in MADS29^OX^ microarray analysis, showing affiliations to key metabolic pathways associated with the MADS29^OX^ phenotype (e.g. flowering, plastid biogenesis, auxin and cytokinin metabolism, and carbohydrate metabolism) for validation by qPCR. The qPCR results for all genes were found to be in good agreement with the microarray results (Fig 5D; see also Supplementary Table S1 for a list of genes showing links to important pathways). Genes such as *AGPase LS1*, *AGPase SS1*, *sucrose synthase*, and *plastidic glucose transporter1* were affected in MADS29^OX^ plants, indicating an imbalance in the carbohydrate metabolism and transport. *OsRR6* and *OsRR9/10* (Jain *et al*., 2006; Hirose *et al*., 2007), which are negative regulators of cytokinin signalling and are cytokinin responsive, were upregulated. Two cytokinin-O-glucosyl transferases, which are involved in inactivating cytokinins, were upregulated (Fig. 5D). Genes involved in auxin signalling, such as *OsSAUR10*, *OsSAUR12*, and auxin-induced protein, were also affected. Some of the prominent gene classes highlighted among the MADS29^KD^ differentially expressed genes included those coding for chloroplast precursors, components of hormone metabolism and signalling, carbohydrate metabolism, LEA (Late Embryogenesis Abundant) proteins, and lipid transfer proteins (Supplementary Table S2). A number of AUX/IAA genes were found to be downregulated, indicating a disruption in the auxin-signalling pathway. Moreover, a few of the cytokinin metabolism genes were also affected. Some of the representative differentially regulated genes identified in the microarray analysis involved in auxin, cytokinin, chloroplast, and carbohydrate metabolism/biosynthesis were selected for validation of microarray results by qPCR. Auxin and cytokinin genes included those coding for OsSAUR21, OsSAUR12, auxin-induced protein, auxin efflux carrier, and cytokinin-N-glucosyltransferase were upregulated, whereas a gene encoding for OsIAA24 was downregulated (Fig. 6E).

## Discussion

### Transcriptional as well as post-transcriptional regulation of *MADS29* expression

*OsMADS29* transcripts have previously been shown to accumulate in a seed-specific manner (Lee *et al*., 2003; Arora *et al*., 2007). Recently, Yin and Xue (2012) and Yang *et al* (2012) found maximum accumulation of *MADS29* transcripts in the nucellus and the nucellar projection as well as the ovule vasculature during early stages of seed development and suggested its involvement in regulating programmed cell death of these cell types. The current data resulting from Western blot and immunolocalization analyses, however, shows that although the *MADS29* mRNA starts accumulating to high levels immediately after pollination, there is at least a 4-day lag in any significant level accumulation of the protein, because the protein is barely detectable at the S2 stage and it is only at the S3 stage (i.e. after 5 DAP) that a MADS29 band is visible in the Western blots. This is indicative of a possible translation level control that prevents *MADS29* transcripts from being translated (Bailey-Serres, 1999; Cheng and Chen, 2004). Furthermore, the lack of corresponding protein in the nucellar projection region, which was shown to accumulate maximum amounts for *OsMADS29* transcripts (Yin and Xue, 2012), further indicates the possibility of a post-transcriptional regulation of *OsMADS29* expression. There are a number of mechanisms that have been shown to affect translation, namely sucrose-mediated translational control, as shown for an *Arabidopsis thaliana* bZIP gene via an upstream open reading frame (uORF) (Hummel *et al*., 2009), and the presence of the light responsive *cis*-element TAGGGTTT in the 5′-untranslated region (Liu *et al*., 2012). Translation is also controlled by miRNA-mediated pathway, where AGO1 associates with miRNA and polysomes to regulate translation (Lanet *et al*., 2009). A preliminary survey of *OsMADS29* has revealed putative binding sites for miR441, miR446, and miR809 in its 3′-untranslated region. Further experiments would prove whether MADS29 translation were under the control of any of the above mentioned mechanisms. The presence of MADS29 protein during late stages, including the mature grain and germination stages (Supplementary Fig. S6), in the absence or low level of transcript suggests that the protein is very stable and has a longer half-life, indicating its importance during seed development.

### MADS29 may have evolved for monocot-specific regulation of embryo and endosperm development

Phylogenetic analysis has revealed that the monocot and dicot B-sister proteins form distinct clades (B-sister monocot subgroup II; Supplementary Fig. S1). This suggests that probably the B-sister monocot subgroup II proteins have acquired a cereal-specific role in seed development after their divergence from dicots (Gallavotti *et al*., 2008; von Behrens *et al*., 2011; Sang *et al*., 2012).

Dicots have an endosperm that disappears with maturation of the embryo, whereas in cereals the endosperm persists and stores starch in amyloplasts (Olsen, 2004). Amyloplasts are modified plastids that constitute major proportion of the endosperm (Thomson and Whatley, 1980) and the process of their differentiation from proplastids is not clearly understood (Yun and Kawagoe, 2009, 2010). Silencing *OsMADS29* leads to reduced grain filling decreased seed length, which resulted in decreased grain weight and hence decreased grain size. The endosperm cells in the knockdown lines were much smaller and were characterized by loosely packed starch grains. MADS29 silencing thus affected the size and number of starch granules along with the size of endosperm cells. A similar, more drastic phenotype of deformed starch granules/amyloplasts and reduced grain filling was observed for *flo2* mutant where expression of many starch metabolism genes was altered (She *et al*., 2010). Previously, Yin and Xue (2012) showed that starch accumulation and quality in the *MADS29* antisense lines was affected along with evidence to show that transcript levels of many starch metabolism genes were changed. It has been shown that altered grain filling can lead to low-quality starch and abnormal starch granules (Wang *et al*., 2008). In MADS29^OX^ lines, components of starch metabolism were upregulated (*Sucrose synthase*) as well as downregulated (*AGPase SS1*, *AGPase LS1*, *SS1*), suggesting that MADS29 is upstream to starch biosynthesis pathway and may affect its components, either directly or indirectly. Both silencing and overexpression of MADS29 affected many plastidic genes, as indicated by the current microarray data, thus implicating it in regulation of plastid differentiation as well. It was also significant to note that a majority of genes common to both MADS29^KD^ and MADS29^OX^ (Supplementary Table S3) exhibited reciprocal expression pattern, thereby suggesting that probably these genes could be direct targets of MADS29.

### Role of MADS29 in maintaining auxin/cytokinin homeostasis

Overexpression of *MADS29* caused severe stunting and other pleotropic effects on growth and development. The MADS29^OX^ phenotype was similar to that seen for certain hormone-related genes. As expected, many auxin and cytokinin signalling and metabolism genes were differentially regulated in MADS29^OX^ as well as MADS29^KD^ lines. MADS29^OX^ plants were also found to have reduced leaf intercalary meristems. The size of the meristem has previously been shown to be affected by the balance of the auxin/cytokinin ratio (Moubayidin *et al*., 2009). In roots, higher levels of cytokinins result in progressive reduction in meristem size by increasing the rate of meristematic cell differentiation, thereby leaving less stem cells, which are insufficient to sustain proper development (Dello Ioio *et al*., 2007, 2008). A recent report on molecular characterization of *dec1* mutants, which exhibited enlarged shoot apical meristem, documented a decrease in cytokinin (such as zeatin and zeatin riboside) levels as well as downregulation of the type-A response regulator genes *OsRR6* and *OsRR9/10* (Itoh *et al*., 2012). In MADS29^OX^ plants also, the observed reduction in the size of intercalary meristem size was accompanied by an increase in cytokinin levels and upregulation of *OsRR6* and *OsRR9/10*. The *OsMADS29* gain-of-function phenotype, therefore, might have resulted due to the influence of MADS29 on genes involved in the cytokinin pathway, leading to an imbalance in cytokinin/auxin ratios and a cytokinin overproduction-like phenotype (Sakamoto *et al*., 2006; Hirose *et al*., 2007).

The role of *OsMADS29* in maintaining hormone homeostasis was further supported by experiments where *MADS29* was overexpressed in BY2 cells. In BY2 cells, the formation of ectopic amyloplasts is associated with exogenous application of cytokinin (Miyazawa *et al*., 1999, 2002; Enami *et al*., 2011), hence giving a lead that probably MADS29 is involved in managing the auxin/cytokinin ratios. These data also hinted towards the involvement of MADS29 in regulating plastid differentiation via cytokinin pathway. The microarray analysis revealed that a large number of chloroplast/plastid precursor proteins were also affected in MADS29^OX^ lines, which may have led to abnormalities in chloroplast development, as indicated by the initial fluorescence (F_0_) of chlorophyll (Supplementary Fig. S7A). Accumulation of MADS29 protein also correlates with the pattern of cytokinin accumulation in rice seeds, which has been directly linked to grain filling (Yang *et al*., 2000, 2002). Cytokinins have been known to influence chloroplast biogenesis and function (Polanska *et al*., 2007; Zubo *et al*., 2008; Brovko *et al*., 2010). In rice, overexpression of *OsRR3* and *OsRR5* has been shown to make the plants insensitive to cytokinin, leading to a reduction in chlorophyll levels (Cheng *et al*., 2010). Mutants impaired in cytokinin perception also show fluctuations in their chlorophyll levels (To *et al*., 2004). The current study observed a reduction in chlorophyll levels in MADS29^OX^ plants (Supplementary Fig. S7B), which may be due to the upregulation of *OsRR6* and *OsRR9/10*. Therefore, the overexpression studies in both rice and BY2 cells and the silencing of *MADS29* in rice suggest that OsMADS29 may have a role in maintaining hormone homeostasis and plastid differentiation during seed development in rice.

**Fig. 7.**
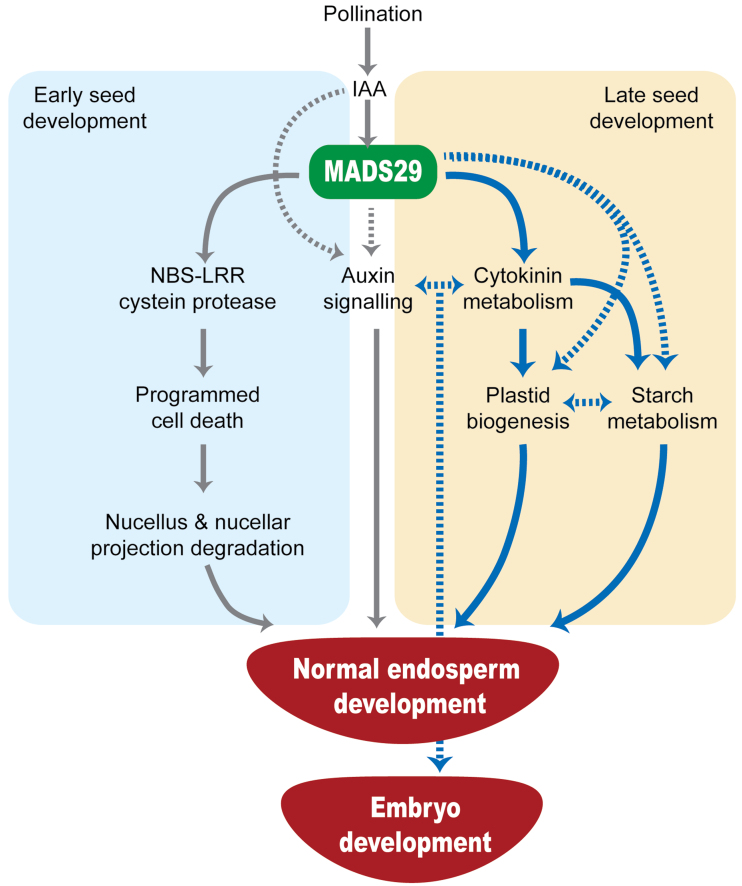
A hypothetical model representing *MADS29* function in seed development. An earlier report by Yin and Xue (2012) described *MADS29*’s role in programmed cell death of maternal tissues, the nucellus, and the nucellar projection during early stages of seed development (blue rectangle). The current data suggest that *MADS29* expression affects cytokinin levels in homologous (rice) as well as heterologous (tobacco BY-2 cell line) conditions during post-5-DAP seed development (peach rectangle). Transcriptome analyses of MADS29^OX^ and MADS29^KD^ plants followed by quantitative PCR-based validation of candidate genes point towards MADS29’s involvement in plastid biogenesis and starch metabolism, either directly or indirectly via the cytokinin pathway. OsMADS29-mediated alterations in auxin and cytokinin homeostasis may also affect cell division and differentiation in the embryo (this figure is available in colour at *JXB* online).

In conclusion, MADS29 seems to belong to a class of cereal-specific transcription factors that primarily targets hormone homeostasis, plastid biogenesis, and starch biosynthesis during seed endosperm development (Fig. 7). The presence of the MADS29 protein in developing embryo tissues and its ability to influence auxin/cytokinin homeostasis may implicate it in the regulation of embryo development as well. Some of the MADS29 target pathways (especially cytokinin biosynthesis) could also exist during vegetative development; therefore, probably the expression of this gene outside its natural domain caused such a drastic phenotype. Taken together, these analyses have revealed important facets of OsMADS29 function in maintaining hormone homeostasis and are also suggestive of its involvement in plastid differentiation and starch biosynthesis during seed development in rice.

## Supplementary material

Supplementary data are available at *JXB* online.

Supplementary Methods S1.

Supplementary Fig. S1. Phylogenetic analysis of MADS-box B-sister proteins.

Supplementary Fig. S2. Enlarged view of a transverse section of immunostained dorsal region of a developing seed (4 DAP).

Supplementary Fig. S3. Responsiveness of MADS29^OX^ roots to auxin.

Supplementary Fig. S4. Reversal of the MADS29-overexpression phenotype by auxin treatment to transgenic BY2 cells.

Supplementary Fig. S5. Cytokinin-responsive genes in MADS29^OX^.

Supplementary Fig. S6. Temporal expression of MADS29 transcript and protein during seed germination.

Supplementary Fig. S7. Initial chlorophyll fluorescence (F_0_) and chlorophyll levels in MADS29^OX^ lines.

Supplementary Table S1. List of genes from selected pathways affected in the MADS29-overexpression lines versus wild-type plants.

Supplementary Table S2. List of genes from selected pathways affected in the MADS29-knockdown lines versus wild-type plants.

Supplementary Table S3. List of genes common to MADS29^KD^ and MADS29^OX^.

Supplementary Table S4. List of primers used in this study.

Supplementary Data
