# Supplementary material for: Functional delineation of rice MADS29 reveals its role in embryo and endosperm development by affecting hormone homeostasis
Source: J Exp Bot. 2013 Aug 8;64(14):4239–53. doi: 10.1093/jxb/ert231 (PMC3808311; doi:10.1093/jxb/ert231)
Supplement: Supplementary Data [file supp_64_14_4239__index.html]

Functional delineation of rice MADS29 reveals its role in embryo and endosperm development by affecting hormone homeostasis — Supplementary Data 

# Functional delineation of rice *MADS29* reveals its role in embryo and endosperm development by affecting hormone homeostasis

## Supplementary Data

Data files

**Files in this Data Supplement:**

- Supplementary Data - Supplementary Data
