# Supplementary material for: Functional delineation of rice MADS29 reveals its role in embryo and endosperm development by affecting hormone homeostasis
Source: J Exp Bot. 2013 Aug 8;64(14):4239–53. doi: 10.1093/jxb/ert231 (PMC3808311; doi:10.1093/jxb/ert231)
Supplement: Supplementary Data [file supp_ert231_ert231_replacement_Supp__Data_714b.pdf]

Functional delineation of *OsMADS29* reveals its role in embryo and endosperm development by affecting hormone homeostasis

Saraswati Nayar[a], Rita Sharma[a,1], Akhilesh Kumar Tyagi[b] and Sanjay Kapoor[a,2]

Supplementary Figures

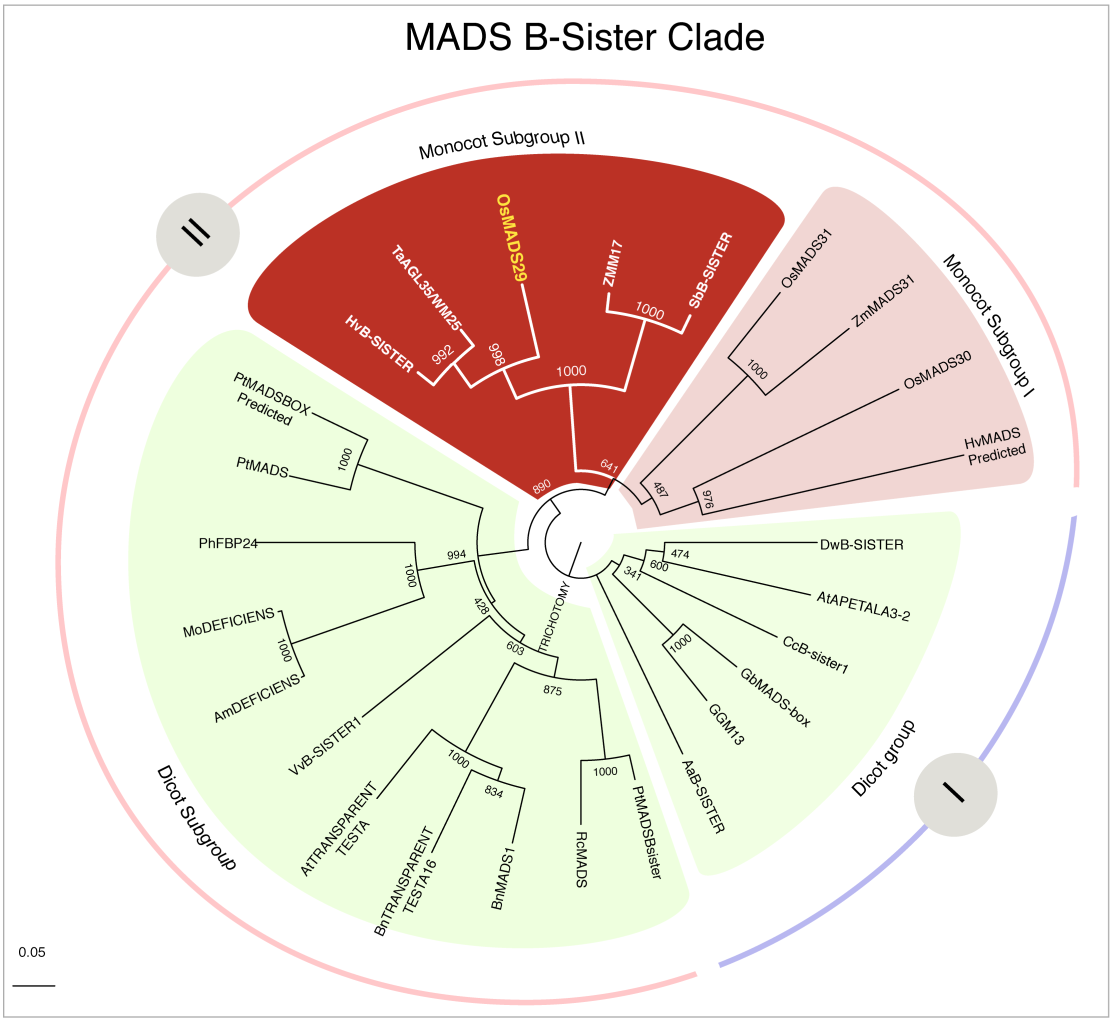

Figure S1. Phylogenetic analysis of MADS box B-sister proteins. B-sister proteins from 13 dicot plants and 5 monocot plants were analysed. These proteins form two major groups; I and II. In Group II there are three subgroups; one for dicots and two for monocot (red and pink) out of which MADS29 (yellow) lies in the Monocot subgroup II (red) along with four other cereal specific B-sister proteins.

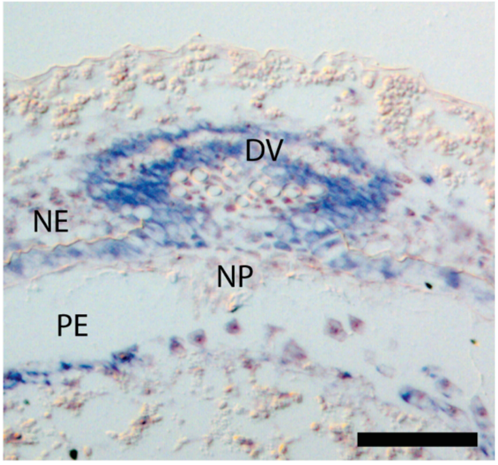

Figure S2. Enlarged view of transverse section of immuno-stained dorsal region of developing seed (4 DAP) showing localization of MADS29 protein in the dorsal vascular bundle (DV), nucellar epidermis (NE) and peripheral endosperm (PE) but no accumulation in nucellar projection (NP). Bar= 50 μm

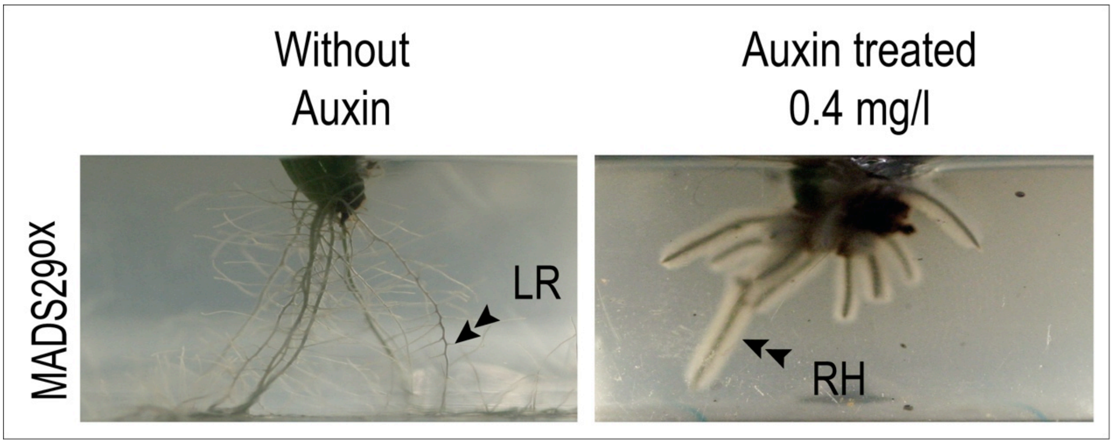

Figure S3. Responsiveness of MADS29^{OX} roots to auxin. Here transgenic roots that were treated with auxin (0.4 mg/l 2,4-D) are showing a typical hairy root and inhibition of lateral root formation indicative of normal auxin response compared to untreated transgenic roots, LR; lateral root, RH; root hair.

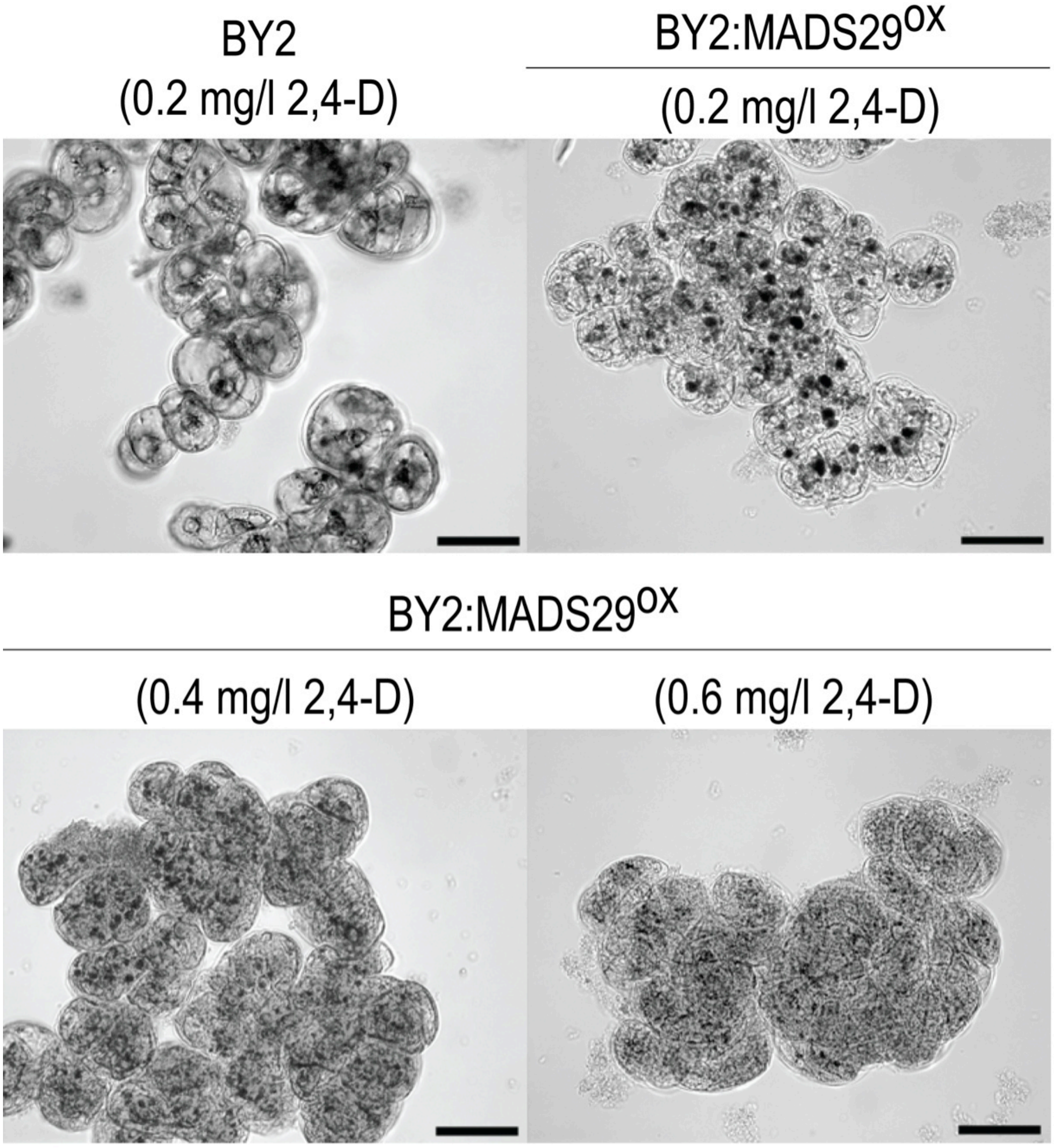

Figure S4. Reversal of MADS29 overexpression phenotype by auxin treatment to transgenic BY2 cells. The BY2-MADS29^{OX} transgenic cell lines were incubated in 0.2 to 0.6 mg/l 2,4-D; Bar = 100 μm.

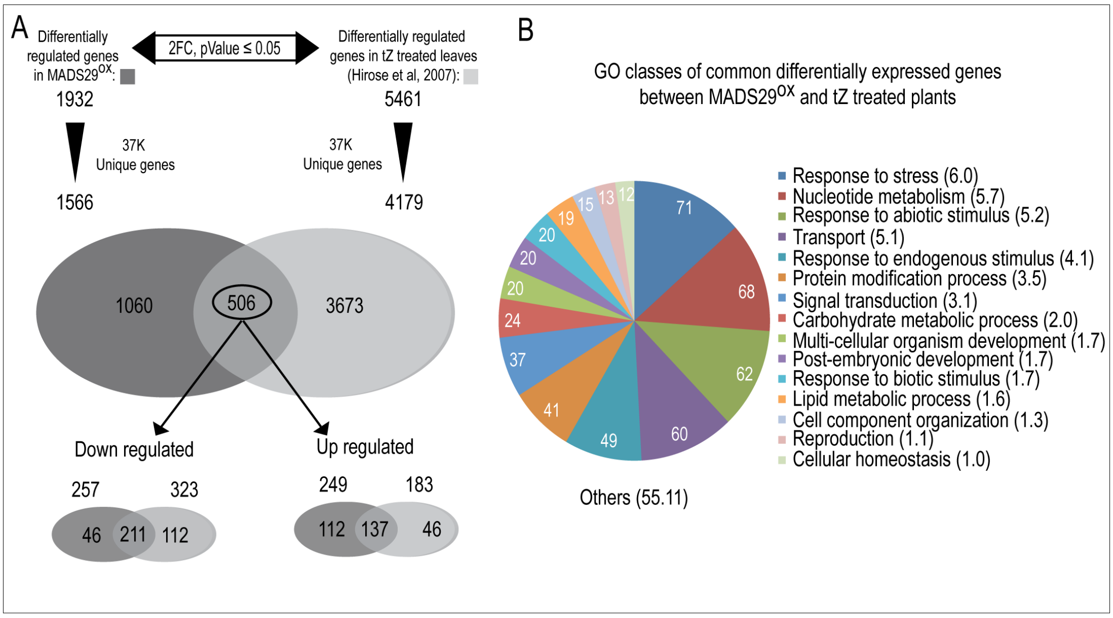

Figure S5. Cytokinin responsive genes in MADS29^{OX} (A) Common differentially regulated genes (2-fold, pValue≤0.05, n=3) in MADS29^{OX} and trans-Zeatin (tZ) treated leaves (Hirose et al. 2007); In MADS29^{OX} lines there are 1932 differentially expressed genes versus WT, out of 1566 are uniquely represented on the Affymetrix Chip, similarly in tZ treated leaves versus wild type there are 5461 differentially expressed genes, out which 4179 are unique. There are 506 genes common between these two datsets out of which 211 genes are commonly down regulated and 137 are commonly up regulated (B) Functional categorization of differentially regulated genes into biological process GO classes in MADS29^{OX} lines; values in brackets and those mentioned on the pie chart are percentages and number of genes represented in each category; categories with <1% share of the differentially expressed genes were not included in the pie chart and are mentioned as 'others'.

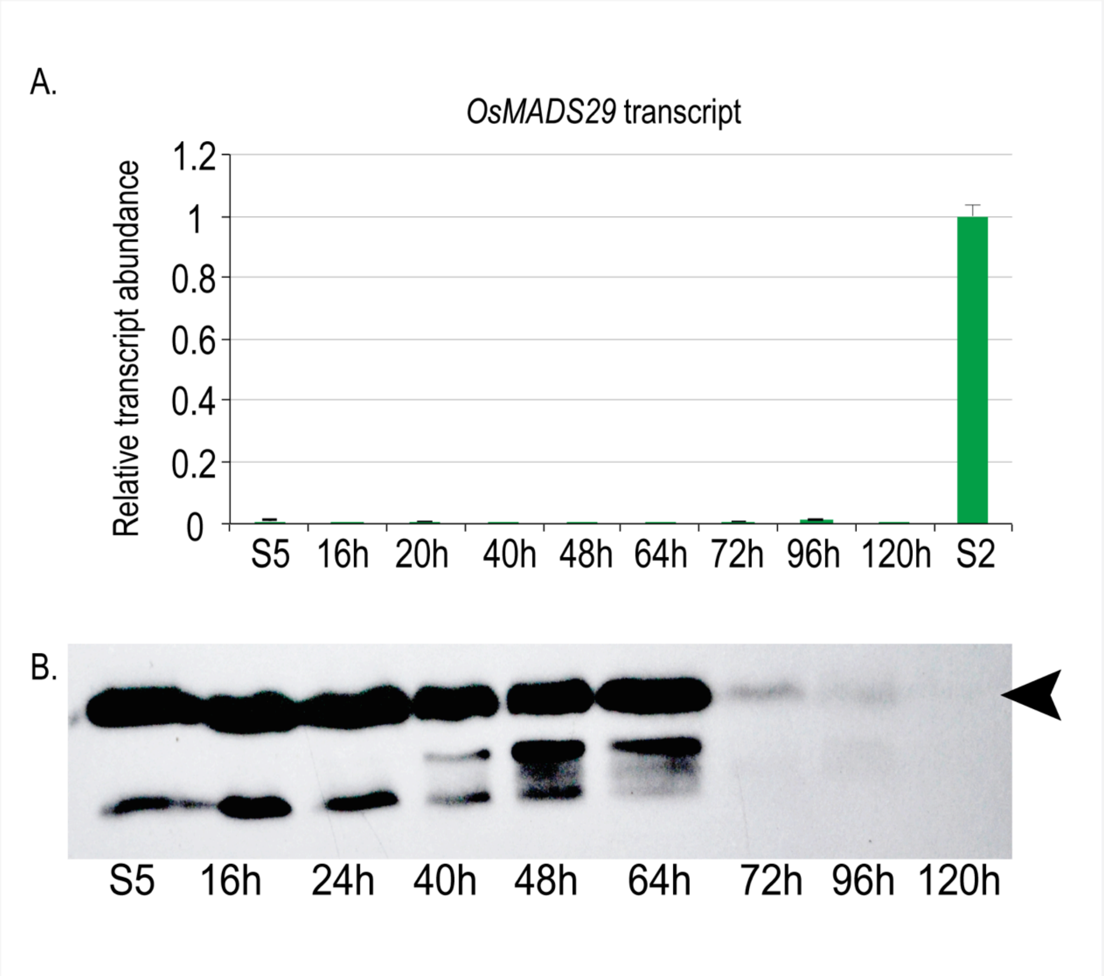

Figure S6. Temporal expression of MADS29 transcript and protein during seed germination. (A) Relative transcript levels of *MADS29* across germination stages (16-120h after imbibition) and seed stages (S2, S5), bar indicates standard error (n=3), (B) Western blot analysis of MADS29 protein using MADS29 antibody across germination stages 16h-120h after imbibition; arrow indicates MADS29 band.

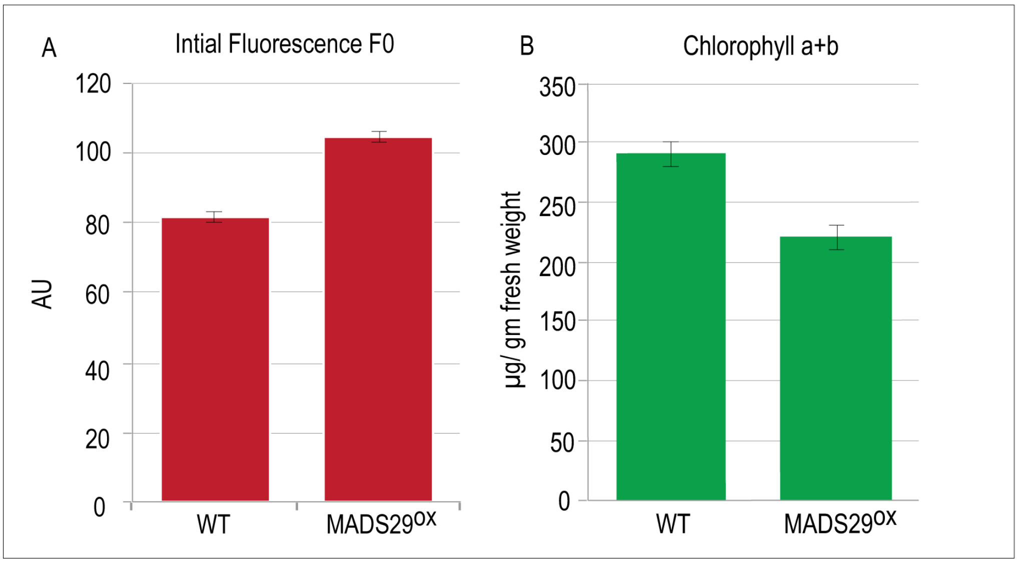

Figure S7. Initial chlorophyll fluorescence (F_0) and chlorophyll levels in MADS29^{OX} lines. (A) Initial chlorophyll fluorescence (F_0) in wild type plants and MADS29^{OX} lines (AU; aribtary unit), bar indicates standard error (n=3). (B) Chlorophyll (a + b) levels in wild type and MADS29^{OX} lines, bar indicates standard error (n=3).

Supplementary Methods S1

Preparation of constructs and plant transformation

A 1325 bp ApaI-EcoRI fragment of *OsMADS29* full-length cDNA KOME clone (AK109522; Rice Genome Resource Center, RGRC, Tsukuba, Japan) was cloned in pBluescript II SK+ (Stratagene, La Jolla, USA). Subsequently, the BamHI-KpnI fragment from pBluescript II SK+ harboring complete *OsMADS29* CDS was transferred to pB4NU binary vector.

For generating RNAi based knockdown (MADS29^{KD}) phenotypes, the *OsMADS29* cDNA sequence was analyzed for siRNA forming potential using “Rational_siRNA_design” software (Reynolds *et al.*, 2004) and unique regions with high score were selected. An identified 204 bp region was amplified using iProof^{TM} DNA polymerase (Bio-Rad, USA) using primers listed in Table S4. The amplicon was initially cloned in an entry vector (pENTR^{TM}) and then in destination vector pANDA; (Miki and Shimamoto, 2004) following Gateway^{TM} cloning strategy (Directional TOPO Cloning kit, and LR clonase Enzyme mix II kit, Invitrogen Inc. USA). The AGL1 strain of Agrobacterium harboring pB4NU-OsMADS29 was used for rice and BY2 cell line transformations to obtain overexpression (MADS29^{OX}) phenotypes, while the one harboring pANDA-MADS29 was used for generating knockdown (MADS29^{KD}) phenotypes. Rice transformation was carried out using PB1 variety of indica rice as previously described (Mohanty *et al.*, 1999).

BY2 cells were transformed by using protocol described earlier (An, 1985) with few modifications. The transgenic calli were selected on LS (Linsnaier and Skoog) medium containing hygromycin (25 mg/l). PCR and GUS staining were used to confirm the transgenic nature of the cells.

Western blot analysis and immunolocalization of MADS29

Total protein was extracted from S1 to S5 seed stages, P1-P6 panicle stages, mature leaf (ML), mature root (MR) and germinating seeds (0—120 h after imbibition) in a minimum amount of 62.5 mM Tris-HCl buffer (pH 6.8) containing 2% (w/v) SDS, 30% (v/v) glycerol, 0.1 M b-mercaptoethanol, 1.5

mM PMSF, and 1 mM EDTA at 4°C (For tissue staging, see Sharma *et al.,* 2012). The homogenate was centrifuged and supernatant was transferred to a fresh tube. Protein was precipitated from this homogenate with chilled acetone containing 10 mM ß-ME. Proteins were pelleted and dissolved in Lamaelli Buffer (0.5 M Tris-HCl, pH 6.8, glycerol, 10% SDS, 0.5% bromophenol Blue, 0.71 M b-mercaptoethanol) and subsequently boiled for 5 min; supernatant was transferred to a fresh tube. Concentration of protein in each extract was determined by Bradford assay (Bradford, 1976) and equal amount of protein (30 μg) was loaded onto a 15% polyacrylamide: SDS gel for electrophoresis at maximum voltage using Mini-PROTEAN Tetra cell apparatus (Bio-Rad, USA). Blotting was performed in a Mini-trans blot apparatus (Bio-Rad, USA) at 100 V for one hour as per manufacturers protocol. Immuno-detection of MADS29 was carried out using antiMADS29 antibody (generated by using a small MADS29 specific peptide; KAPAYYGEESS; Abexome, Bangalore) after 1:5000 dilution and ECL Plus kit (Amersham, GE Healthcare Life Sciences) as per manufacturers protocol.

For in situ immuno-localization of MADS29, rice seeds were fixed in 4% paraformaldehyde: 0.5% glutaraldehyde for 24 h at 4°C and then dehydrated in a graded ethanol (30% to 100%) series followed by a tertiary-butanol (25% to 100%) series, before placing in paraplast plus (Sigma Aldrich). For 1-8 DAP seeds transverse sections (12 μm) of the wax embedded seed tissue were cut using Leica RM2245 rotary microtome and were placed on Poly-L lysine coated slides (Polysciences Inc.) For 14 and 27 DAP seeds free hand longitudinal sections were done on fixed seeds and treated in the same way as the sections. Sections were deparaffinized and incubated in blocking buffer (1XPBS, 5% fat free milk) for 2 h and then in 1:333 dilution antiMADS29 primary antibody in 1×PBS, 0.1% Tween20, 5% fat-free milk), overnight at 4°C. The slides were washed with 1×PBS, 0.1% Tween20 and then incubated in secondary antibody (anti-IgG rabbit antibody conjugated to Alkaline phosphatase) for 2 h. The slides were washed again with 1×PBS, 0.1% Tween20 and incubated in NBT/BCIP solution (Amresco Inc.) till there was

visible colour in the sections. Sections were mounted in DPX mounting medium (Qualigens chemicals, Fisher Scientific) and observed and photographed under a compound microscope (DM 5000B, Leica Gmbh, Wetzlar, Germany).

RNA isolation

Total RNA was isolated from all tissues except seed using TRIzol reagent, following manufacturer's instructions as described earlier (Chomczynski and Sacchi, 1987). Due to high carbohydrate content, RNA from seed samples was isolated using the method described earlier (Singh *et al.*, 2003). Quality of RNA samples was assessed using Bio-analyzer (Agilent technologies, USA) as per manufacturer's protocol. The RNA samples with O.D. ratios at 260/280 nm in the range of 1.9–2.1 and at 260/230 nm in the range of 2.0–2.3 (ND-1000 Spectrophotometer, ThermoScientific, USA) were used for microarray and real-time Q-PCR experiments.

Transcriptome analysis of MADS29^{OX} and MADS29^{KD} rice transgenics

500 ng of total RNA isolated from MADS29^{OX} and WT leaves (Leaf blade, Lamina joint, leaf sheath) and of MADS29^{KD} and WT S3-stage seeds were amplified and labeled using a 3´IVT Express kit (Affymetrix, CA, USA). Target preparation, hybridization, washing, staining and scanning of the chips were done according to the manufacturer's protocol. Affymetrix GeneChip Command Console® 3.0 (AGCC) software was used for washing and staining of the chips in a Fluidics Station 450 (Affymetrix, CA, USA) and scanned with a Scanner 3300 (Affymetrix, CA, USA). Three biological replicates processed for each stage with overall correlation coefficient values of ≥ 0.95 were used for the final data analysis. Differential expression analyses for both over-expression and knock-down lines were performed on GC-RMA normalized datasets with a fold-change cut off of 2-folds at p-value≤ 0.05 using ArrayAssist 5.0.0 microarray data analysis software (Stratagene).

The microarray data have been deposited into the Gene Expression Omnibus database (GSE42029 and GSE42028; http://www.ncbi.nlm.nih.gov/geo).

Quantitative PCR analysis

In case of rice, the cDNA for Q-PCR were synthesized using the same RNA samples that were used for microarray analyses, while for BY2 WT and MADS29^{ox} lines good quality RNA samples conforming to O.D. value standards described above were used for cDNA synthesis. Real-time PCR primer designing, reactions were carried out as described previously (Arora *et al.*, 2007). The relative abundance of transcript was calculated as previously described (Derveaux *et al.*, 2010; Livak and Schmittgen, 2001). Primers used in the experiment are listed in Table S4.

Phylogenetic analysis

B-sister MADS box proteins were identified by BLASTp with OsMADS29 protein sequence as the query, the resulting complete protein sequences B-sister proteins were aligned using ClustalX (version 1.83) program to check the number of groups formed. An un-rooted neighbor joining (Saitou and Nei, 1987) phylogenetic tree was constructed in ClustalX with default parameters (Arora *et al.,* 2007). Bootstrap analysis was performed using 1000 replicates. The tree thus obtained was viewed using TREEVIEW software (Arora *et al.*, 2007; Page, 1996)

Chlorophyll measurement

Chlorophyll was extracted from MADS29^{OX} and WT leaves using 100% acetone according to the protocol described earlier (Polanska *et al.*, 2007). The total chlorophyll content (a+b) was calculated from absorbance taken at 662 nm and 645 nm according to equations described earlier (Lichtenthaler and Wellburn, 1983). Initial chlorophyll fluorescence (F_0) of WT and MADS29^{OX} leaves was measured using a PAM fluorometer (PAM-210; Heinz Walz GmbH, Effeltrich, Germany) as per manufacturer's protocol.

Hormone extraction and ultra high performance chromatography

50 mg plant material was ground in liquid nitrogen; MADS29^{ox} and WT leaf samples were extracted with 80% methanol (Merck, HPLC grade), for 12 h at 4°C in the dark. The methanol fractions of the extracts were passed twice

through a C18 Sep-Pak cartridge (Waters Ass., Milford, MA). The eluates were evaporated completely in vacuum, at 40°C and dissolved in 200 μl absolute methanol. 5 μl of crude extract was subjected to Ultra high performance chromatography (Waters, USA) with the following parameters: C8 column, 30% methanol: 70% water, Flow rate; 0.159 ml /minute, Temperature; 12˚C, Total run time; 10 minutes. The chromatogram of transgenic and wild type crude hormone extract was compared to standards profile (Adenosine (Sigma Aldrich Cat No. A9251-1G), trans-zeatin (Sigma Aldrich Cat No. Z0876-5MG), trans-zeatin riboside (Sigma Aldrich Cat No. Z0375-5MG), Indole acetic acid (Sigma Aldrich Cat No. I3750-5G-A).

References S1

An G. 1985. High efficiency transformation of cultured tobacco cells. *Plant Physiology* **79**, 568-570.

Arora R, Agarwal P, Ray S, Singh AK, Singh VP, Tyagi AK, Kapoor S. 2007. MADS-box gene family in rice: genome-wide identification, organization and expression profiling during reproductive development and stress. *BMC Genomics* **8**, 242.

Bradford MM. 1976. A rapid and sensitive method for the quantitation of microgram quantities of protein utilizing the principle of protein-dye binding. *Analytical Biochemistry* **72**, 248-254.

Chomczynski P, Sacchi N. 1987. Single-step method of RNA isolation by acid guanidinium thiocyanate-phenol-chloroform extraction. *Analytical Biochemistry* **162**, 156-159.

Derveaux S, Vandesompele J, Hellemans J. 2010. How to do successful gene expression analysis using real-time PCR. *Methods* **50**, 227-230.

Lichtenthaler HK, Wellburn AR. 1983. Determinations of total carotenoids and chlorophylls a and b of leaf extracts in different solvents. *Biochemical Society Transactions* **603rd Meeting, Liverpool**, 591-592.

Livak KJ, Schmittgen TD. 2001. Analysis of relative gene expression data using real-time quantitative PCR and the 2(-Delta Delta C(T)) Method. *Methods* **25**, 402-408.

Miki D, Shimamoto K. 2004. Simple RNAi vectors for stable and transient suppression of gene function in rice. *Plant and Cell Physiology* **45**, 490-495.

Mohanty A, Sharma NP, Tyagi AK. 1999. *Agrobacterium*-mediated high frequency transformation of an elite indica rice variety Pusa Basmati and transmission of the transgene to R2 progeny. *Plant Science* **147**, 125-135.

Page RD. 1996. TreeView: an application to display phylogenetic trees on personal computers. *Computer Applications in the Biosciences* **12**, 357-358.

Polanska L, Vicankova A, Novakova M, Malbeck J, Dobrev PI, Brzobohaty B, Vankova R, Machackova I. 2007. Altered cytokinin metabolism affects cytokinin, auxin, and abscisic acid contents in leaves and chloroplasts, and chloroplast ultrastructure in transgenic tobacco. *Journal of Experimental Botany* **58**, 637-649.

Reynolds A, Leake D, Boese Q, Scaringe S, Marshall WS, Khvorova A. 2004. Rational siRNA design for RNA interference. *Nature Biotechnology* **22**, 326-330.
Saitou N, Nei M. 1987. The neighbor-joining method: a new method for reconstructing phylogenetic trees. *Molecular Biology and Evolution* **4**, 406-425.
Singh G, Kumar S., Singh P. 2003. A quick method to isolate RNA from wheat and other carbohydrate-rich seeds. *Plant Molecular Biology Reporter* **21**, 93a-f.
